# Design and psychometrics of the family caregivers burnout inventory of older adults with chronic disease

**DOI:** 10.3389/fpubh.2024.1449273

**Published:** 2024-10-03

**Authors:** Kataneh Farokhmanesh, Abbas Shamsalinia, Mohammad Reza Kordbageri, Kiyana Saadati, Reza Ebrahimi Rad, Fatemeh Ghaffari

**Affiliations:** ^1^Student Research Committee, Nursing Care Research Center, Health Research Institute, Babol University of Medical Sciences, Babol, Iran; ^2^Nursing Care Research Center, Health Research Institute, Babol University of Medical Sciences, Babol, Iran; ^3^Department of Statistics and Mathematics, Shahid Beheshti University, Tehran, Iran; ^4^Student Research Committee, Mazandaran University of Medical Sciences, Ramsar, Iran; ^5^Department of Medicine, Islamic Azad University Tonekabon Branch, Tonekabon, Iran

**Keywords:** chronic disease, older adults, family caregivers, burnout measurement, psychometric evaluation, informal caregiving

## Abstract

**Background:**

Identifying the hidden and pervasive phenomenon of burnout among family caregivers of older adults with chronic disease requires the use of a valid and reliable tool tailored to the cultural structure of the target community. Therefore, the present study aimed to design and psychometrically evaluate the family caregivers burnout inventory (FCBI) of older adults with chronic disease.

**Methods:**

This research is a sequential exploratory mixed-methods study focused on instrument design, conducted in Iran in 2024. The study employed classical theory, involving three stages to create a valid instrument: item design using inductive (semi-structured face-to-face interviews with 13 caregivers) and deductive (literature review) methods, tool design, and tool evaluation using face validity, content validity, construct validity [exploratory factor analysis (EFA) (*N* = 297) and confirmatory factor analysis (297 participants)], convergent validity, and reliability (internal consistency and stability). Data were analyzed using AMOS version 24 and SPSS version 26.

**Results:**

Based on qualitative findings, participant quotes, and item adoption from other studies, a pool of 228 items was designed using a 5-point Likert scale (always to never). After several stages of review by the research team, 102 items remained. Following face validity (2 items), content validity (46 items), and construct validity (23 items due to factor loadings less than 0.4 and cross-loadings), 71 items were eliminated, leaving 31 items. EFA results indicated that the family caregiver’s burnout construct of older adults with chronic diseases comprises six factors include; “neurosis,” “feeling victimized,” “extreme fatigue and helplessness,” “neglect or abuse of self and others,” “adaptation deficiency” and “emotional separation” explaining 52.93% of the total variance. The fit indices showed acceptable model fit with the data. In this study, composite reliability and average variance extracted (AVE) for the six factors were greater than 0.7 and 0.5, respectively, and the (AVE) for each factor was higher than the composite reliability. Cronbach’s alpha coefficient for the entire scale was 0.975, and there was a significant correlation between test and retest scores (*p* < 0.001).

**Conclusion:**

FCBI demonstrates suitable validity and reliability and can be used in various settings by health service providers to identify symptoms of burnout in family caregivers.

## Background

Older adults, due to their life conditions and the aging process, are susceptible to various chronic diseases, including high blood pressure, diabetes, heart diseases, and numerous psychological disorders, which significantly impact economic, social, and cultural aspects (1). Most of them face multiple chronic conditions, meaning they suffer from two or more chronic diseases, a phenomenon that has increased among adults worldwide over the past 20 years (2). Between 55 to 98% of the older adults are dealing with such conditions (3). This group of older adults, due to their multiple chronic conditions, must frequently visit health service providers for diagnostic services or follow-up treatments. Many of them have a low quality of life or are hospitalized due to medical needs. Older adults with chronic diseases, due to the development of care needs in various health dimensions, may face challenges in self-care interventions across different aspects of their personal and social lives. These individuals often suffer from physical and psychological problems, leading to further complexity in self-care (3, 4). Therefore, many of them require assistance from formal and informal caregivers to meet their growing needs in various health dimensions (5). Existing research evidence shows that the older adults generally prefer to receive home care, which aligns with government programs to delegate caregiving responsibilities to families at home (6, 7).

The complexity of care required for older adults with chronic diseases necessitates a shift beyond acute care to long-term home care (8). Nowadays, the formal medical role has shifted from healthcare professionals such as nurses and doctors to family caregivers. Family caregivers are responsible for providing most caregiving tasks, ranging from personal care to medical care, for older adults with chronic diseases (6). These caregivers face a wide range of demands over a prolonged caregiving period, indicative of chronic stress (9). They encounter various pressures, such as assisting patients with daily living activities, managing multiple appointments with healthcare specialists in different locations, helping the older adults adhere to multiple and complex medication regimens, and dealing with changes in their roles and responsibilities (10). Consequently, these individuals experience caregiving-related burden and live under stressful conditions, which has become a growing concern (7). Research indicates that caregivers experience an excessive prevalence of stress-related emotional burdens, limitations in social/recreational activities, decreased appetite and sleep, and an increased risk of mortality and psychological disorders compared to non-caregivers (11, 12). Cohen and colleagues note that such caregivers may no longer be able to continue their caregiving role, and if other family members or friends cannot assume the caregiving responsibility or if there is no support from formal or informal care systems, they may face burnout syndrome (13).

Burnout syndrome can manifest in two forms: psychological (low self-esteem, fatigue, anxiety, hopelessness, lack of concentration, and clinical manifestations such as headaches, insomnia, pain, and gastrointestinal issues) and behavioral (caffeine consumption) (14). The emergence of some burnout symptoms may interfere with the quality of care and lead to early or repeated hospitalization of the older adults. It may also result in social isolation, living under prolonged stressful conditions, increased biological vulnerability, a higher risk of developing psychological disorders, and an increased incidence of physical problems in caregivers (such as high blood pressure, increased stress-related hormones, immune system suppression, severe depression, and fatigue) (15, 16). Therefore, developing and implementing programs for caregivers can lead to high-quality care and reduce caregiving outcomes such as caregiving burden and burnout (17), thereby facilitating continuous care. Achieving this goal can be obtained through evidence-based data (18).

Since caregivers accompany the older adults to appointments with healthcare providers, including geriatric nurses, nurses play a key role in identifying caregiver burnout and can help prevent this phenomenon by evaluating and providing sufficient support to caregivers. Using valid and reliable tools to identify the symptoms and signs of burnout syndrome in informal caregivers of patients with chronic diseases can facilitate timely interventions by nurses and other healthcare providers to assist informal caregivers (19). Existing tools primarily focus on caregiver burden and do not specifically address the various aspects of caregiver burnout. These tools may be insufficient for accurately and comprehensively identifying burnout symptoms in family caregivers. Informal (family) caregivers work in environments and conditions that differ from formal work settings. Currently, screening for burnout syndrome is conducted using several tools, some of which are widely used in research. These tools include the Maslach Burnout Inventory (MBI) (20), Pines’ Burnout Measure (BM) (21), Psychologist Burnout Inventory (PBI) (22), Oldenburg Burnout Inventory (OLBI) (23), burnout potential inventory (24), Granada Burnout Questionnaire (25), Copenhagen Burnout Inventory (CBI) (26), Informal Caregiver Burnout Inventory (ICBI) (27), and the Informal Caregiver Burnout Scale (ICBS) (28).

The results of a systematic review (2021) that examined the psychometric indicators of burnout tools across 6,541 studies showed that 15 studies used the MBI to measure burnout. In contrast, BM, PBI, OLBI, and CBI were each used in only one study. The findings indicated that the OLBI had the most comprehensive validity, followed by the CBI, MBI, BM, and PBI. When examining the precision of these tools in interpreting results, the greatest discrepancies were observed for the MBI (27%), BM (25%), and CBI (17%). No discrepancies were found for the PBI and OLBI. The quality of evidence for content validity and psychometric properties was moderate for the OLBI and CBI. However, for the MBI, BM, and PBI, these indices were reported as very low (29).

Additionally, it should be noted that all these tools, except for the ICBI (27) and the ICBS (28), were designed to measure occupational burnout among formal caregivers or health team members. These tools primarily focus on occupational and workplace aspects within healthcare service delivery systems, and none of the mentioned tools specifically measure the burnout construct in family caregivers. Although these tools, especially the MBI, have been used in numerous studies to measure burnout among family caregivers of the older adults (15, 30–32), their results do not seem to have the necessary validity. Perhaps the most prominent difference between informal and formal caregiving lies in the environment and organizational structure. Formal employment involves workplace factors such as colleagues, supervisors, support units, corporate guidelines, agreed working hours, contracts, holidays, and even days off or sick leave. These factors do not directly impact the structure of caregiver burnout. Therefore, a specialized tool is necessary to measure burnout among informal caregivers (27). Although the ICBI and the ICBS are designed to measure the burnout construct among family caregivers, both are unidimensional and cannot comprehensively measure the dimensions of family caregiver burnout. Moreover, attention to the health of family caregivers has become one of the priorities of research projects worldwide to achieve this goal (33). To this end, designing culturally appropriate tools based on standard instrument development protocols to measure various health dimensions by researchers has seen significant growth (34). They usually do not benefit from organizational supports such as vacations, insurance, and colleague support, which can lead to increased stress and burnout.

## Objectives

Therefore, designing an appropriate tool to identify and manage caregiver burnout can help improve the quality of life for both caregivers and patients. To develop and implement supportive and intervention programs to reduce caregiver burnout, accurate and reliable data is needed. Existing tools may not fully and accurately provide the necessary information. Designing a new and specific tool can help collect better and more precise data. Existing tools may not fully align with the culture and social conditions of the target country. Designing a localized tool can help better and more accurately identify caregiver burnout in family caregivers. Due to the lack of access to a comprehensive and specific tool for measuring the burnout construct among family caregivers, the researchers in this study aimed to design and psychometrically evaluate the family caregivers burnout inventory (FCBI) of older adults with chronic disease.

## Materials and methods

### Study design, participants, and procedure

The present research is a sequential exploratory mixed-methods study focused on instrument design, where the researchers collect and analyze data using both qualitative and quantitative approaches (35). In this study, the researchers first collected and analyzed qualitative data and then used the information obtained to determine how to collect the quantitative data in the subsequent stage. The study population consisted of all family caregivers of older adults with chronic diseases. Sampling was conducted in two stages: qualitative sampling using purposive sampling and quantitative sampling using convenience sampling.

The researcher visited the health information management units of hospitals affiliated with Babol University of Medical Sciences, Iran, and compiled a list of older adults with chronic diseases admitted to various departments, including CCU, internal medicine, gastroenterology, cardiology, infectious diseases, orthopedics, kidney transplantation, dialysis, neurology, hematology, and rheumatology. Additionally, the researcher obtained a list of older adults attending the associated clinics. The researcher then explained the research objectives to the caregivers who were present as patient companions in the hospitals and clinics. After assessing the inclusion criteria and obtaining consent, the researcher collected their phone numbers and provided them with the research tools via social media for completion. The inclusion criteria were: being the primary caregiver for the patient for at least 1 year, being 18 years or older, being a first-time caregiver, having access to the internet and a smartphone, and being a relative of the patient by blood or marriage. The exclusion criteria were the unwillingness to continue participating in the research or failure to complete the online questionnaire.

### Steps in designing and psychometric evaluation of the FCBI of older adults with chronic disease

Most tools used in nursing research are developed based on classical test theory; hence, this study also utilized this theory. There are three stages in creating a valid instrument: item design, tool design, and tool evaluation (36).

#### Item design

This stage involves the creation of unique items. Two approaches exist for item creation: inductive and deductive. Instrument designers must decide which of these two methods is more appropriate for their study (37). In this study, to design the FCBI of older adults with chronic Disease, both themes identified from the qualitative study and reviews of existing literature and tools were used to ensure the designed tool comprehensively covers the dimensions of family caregiver burnout.

### Qualitative stage/inductive method

The interviews were coded by the research team. The analyses were conducted manually, and used conventional content analysis for data analysis. In this stage, a qualitative content analysis with a conventional approach was used. Qualitative content analysis allows researchers to interpret the authenticity and truthfulness of data subjectively but scientifically (38, 39). Data collection in this study was conducted through individual, semi-structured, and in-depth interviews. The interview questions were developed based on a review of the literature and the initial experiences and feedback from the caregivers. The number of 13 caregivers was chosen due to data saturation. The interviews were conducted by an expert (the corresponding author) using in-depth, face-to-face interviews to collect data until saturation was reached. Before starting the interview, the interviwer introduced themselves and explained the study’s objectives to the participant. Then, the interview began by establishing rapport and gaining the participant’s trust. At the beginning of each interview, questions such as “Please introduce yourself” and “How are you?” were asked to create interaction between the researcher and the participant, aiding in trust-building and starting the interview. Subsequent questions were asked based on the interview guide, including questions like “Please tell me about your experience in caring for a patient with a chronic disease,” “What challenges did you face while providing care?” and “What were the physical, mental, emotional, and social outcomes for you as a caregiver of a patient with a chronic disease?” Additionally, as needed, exploratory questions such as “What do you mean?” and “Could you please explain to me more?” or “How did you feel about this?” were used during the interviews. At the end of each interview, participants were asked if they had any additional comments and were informed about the possibility of future interviews. All participants provided their phone numbers so follow-up interviews could be scheduled if necessary or for verifying their statements after transcription. A total of 13 in-depth individual interviews were conducted with 13 participants, with each session lasting between 35 to 72 min. For qualitative data analysis, a conventional qualitative content analysis method was used. The interviews were coded by the research team. The analyses were conducted manually, and used conventional content analysis for data analysis. To achieve accurate and reliable information, a systematic and transparent seven-step process suggested by Graneheim and Lundman (40) was employed for data processing. The qualitative content analysis process included, the preparing the data, coding the text, reviewing codes against the text,categorizing and developing categories based on similarities and appropriateness, reviewing categories and comparing them again with the data to ensure the robustness of the codes, identifying themes through careful and thorough reflection and comparing them with each other and reporting the findings. An example of the item design process is presented in Table 1.

**Table 1:** Sample of item formation in the qualitative section.

Quote	Code	Initial category	Item
“I want to go to my mother’s house now. I miss her. I want to hug her. I cannot leave the house. If I want to do something for myself, I cannot. My life is devoted to my spouse.”	-Devoting life to caregiving -Lack of time for personal needs -Providing care while being chronically ill -Fighting with oneself -Complaining about fate and current conditions -Self-blame -Feeling defeated in life -Transforming care into duty -Not receiving adequate appreciation for caregiving -Changing identity	-Feeling victimized -Feeling deprived -Self-blame -Feeling unrecognized	-"I feel like a victim because my needs get lost among the many needs of the care recipient or others.” -"The pressure of caregiving made me constantly think that I do not deserve this situation.” -"I am upset that caregiving has become my duty.” -"My caregiver identity has overshadowed my identity as a man / woman, and it upsets me.”
“I tried hard to take care of myself too. Despite being ill sometimes, I did not say I was sick. I could not take care of myself. I have high blood pressure and diabetes. I am also struggling with illness. I have become a victim in this situation.”			
“I argue with myself about this fate I have. What kind of life is this? What is this?! What sin or crime did I commit to endure this?”			
“I want my spouse to appreciate the things I do. It feels like this work has become my duty. It bothers me. I’ve lost my identity. I feel defeated.”			

### Ensuring validity and accuracy of qualitative findings

In this study, Lincoln and Guba’s parallel criteria were used to ensure the validity and accuracy of the qualitative findings: credibility, dependability, transferability, and confirmability (41). Several measures were taken to enhance credibility, including purposeful sampling with maximum diversity (selecting participants with varying occupations, genders, ages, number of children, durations of caregiving and relationships with the older adult), collecting sufficient data, selecting appropriate meaning units, explaining how categories and themes were formed, allocating sufficient time for data collection, and continuous and iterative engagement with the data. Additionally, the findings were checked with the participants. For this purpose, the transcriptions of some participants’ interviews were returned to them after coding to verify whether the codes assigned by the researcher matched their intended meanings. In some cases, participants indicated that their intended meaning differed from the researcher’s interpretation, and the codes were revised accordingly. Seeking consensus within the research team and conducting external audit reviews were also employed to control the credibility of the data. The researcher (first author) made an effort to ask all participants questions within the same domains during the interviews. All individual interviews were recorded and transcribed verbatim. The researcher’s decisions and activities regarding data collection and analysis were fully and continuously documented. Additionally, initial codes derived from participants’ experiences, examples of how themes were extracted, and excerpts from interviews for each theme were provided. An audit trail was also employed to ensure dependability. The researcher endeavored to minimize the influence of their assumptions in the data collection and analysis process.

### Literature review/deductive method

For the design of the items in this study, relevant articles were reviewed using various databases and scientific search engines, including Scopus, PubMed, Proquest, Iranmedex, Elsevier, and Medlib. The search was conducted using the burnout in informal caregivers, burnout in family caregivers, caregivers burnout questionnaire, caregivers burnout scale, caregivers burnout list, caregiver burnout tool, older adults with chronic diseases, informal caregivers burnout inventory and informal caregivers burden experience keywords without time restrictions.

## Instrument design

In this stage, the researcher needs to employ a set of items determined for the construct or constructs of the instrument and evaluate how well these items meet the expectations of the instrument’s structure (36). When designing an instrument, items are generated for respondents to answer. These responses are then converted into numerical form and statistically analyzed (42). Hence, the number of items in each scale and the length of the scale affect the quality of the responses (36). When designing a new measurement instrument, it is essential to determine the type of scale and response format to be used (42). In social sciences, the Likert format is the most common scale used for designing attitudinal scales (43). In the instrument designed for the present study, a five-point Likert scale ranging from “always” to “never” was used. The pilot study indicated that this number of options increased respondent accuracy and the receipt of genuine responses. In this tool, the option “always” was scored five, and “never” was scored one. The pilot testing of the instrument involved multiple readings of the items by the researcher, asking family caregivers to read the items in the presence of the researcher to identify any unclear items or words (10 participants in the face validity stage), and requesting feedback from the experts participating in the psychometric panel (10 participants). Throughout the psychometric process, the researcher consistently revised and improved the items. The initial instrument, along with questions related to demographic information, consisted of 16 and 54 items, respectively. Efforts were made to ensure that the items were as straightforward and clear as possible, with an estimated completion time of 10 to 15 min.

## Instrument evaluation

To determine the psychometric properties of the FCBI of older adults with Chronic Disease, face validity, content validity, construct validity, and reliability were assessed.

### Face validity

In this study, both qualitative and quantitative methods were used to determine face validity. In the qualitative method, the items were initially presented to 10 experts (faculty members in nursing, clinical psychology, and psychiatry), who were asked to comment on the difficulty level, relevance, and ambiguity of the items. Subsequently, quantitative face validity was evaluated using the item impact method. For this purpose, 10 experts (the same individuals invited for qualitative face validity assessment) were asked to rate the importance of each item based on their experiences using a five-point Likert scale (from “very important” to “not important at all”) (44).

### Content validity

Content validity in this study was assessed using both qualitative and quantitative methods. For qualitative content validity, the same 10 experts (who were also involved in the face validity assessment) were asked to provide feedback on the grammar, appropriateness of wording, and placement of items. The research team reviewed their feedback and made the necessary revisions. For quantitative content validity, the content validity ratio (CVR) and content validity index (CVI) were calculated (44).

### Content validity ratio (CVR)

To calculate the CVR, 10 experts were asked to rate each item on a three-point scale (not necessary to necessary) in terms of its necessity. According to Lawshe’s table, a score of 0.62 or higher was considered the criterion for retaining an item (45). The result obtained was compared with the criterion value in Lawshe’s table (0.62) (46) based on the number of the experts (47). If the obtained value was greater than the table value, it indicated that the item was statistically significantly necessary for the questionnaire (*p* < 0.05) (45). In this study, the CVRstrict method was used, meaning that only items deemed necessary were included in the CVR calculation formula (48). The CVR was calculated using the following formula:

$$CVR\;\mathrm{strict}=\frac{n_{e}-N/2}{N/2}$$

Where n_e_ is the number of experts who rated the item as essential, and N is the total number of experts. The CVR value ranges from −1 to +1. A CVR < 0 indicates that fewer than half of the experts believe the item is essential. A CVR of 0 means exactly 50% of the experts consider the item essential, and a CVR > 0 means more than 50% of the experts find the item essential (49).

#### Content validity index (CVI)

To evaluate the content validity index, experts were asked to assess each item on a four-point Likert scale for relevance, clarity, and simplicity. Experts rated the relevance of each item from 1 “not relevant,” 2 “somewhat relevant,” 3 “relevant,” and 4 “completely relevant.” Simplicity was rated as 1 “not simple,” 2 “somewhat simple,” 3 “simple,” and 4 “completely simple.” Clarity was rated similarly: 1 “not clear,” 2 “somewhat clear,” 3 “clear,” and 4 “completely clear” (50). An item was accepted if it achieved a CVI score higher than 0.79 (51).

### Construct validity

In this study, construct validity was assessed using both exploratory factor analysis (EFA) and confirmatory factor analysis (CFA). A sample of 596 participants were used for constant validity (297 participants for EFA and 297 other participants for CFA).

#### Exploratory factor analysis (EFA)

To explore the internal relationship between variables and identify clusters of variables with the highest correlations, EFA was conducted using the maximum likelihood (ML) method (52). Before extracting the factors, the Kaiser-Meyer-Olkin (KMO) test for sample adequacy and Bartlett’s test of sphericity were used to ensure that the items were suitable for principal component analysis (53). A sample of 297 participants was used for EFA.

#### Confirmatory factor analysis (CFA)

In CFA, the researcher specifies the number of factors, the variables that reflect these factors, and whether the factors are related (54). A descriptive cross-sectional study was conducted on a sample of 297 participants to perform CFA.

After examining the correlations between factors and identifying the factors in the first-order CFA, second-order CFA was conducted using structural equation modeling to confirm whether the identified factors constituted the construct of family caregiver burnout and to determine the contribution of these factors to the construct.

### Data distribution, outliers, and missing data

Univariate and multivariate data distributions were examined separately to check for normal distribution and outliers. Multivariate outliers were identified using Mahalanobis distance (*p* < 0.001) and multivariate skewness was examined using Mardia’s coefficient (above 20) (55). The percentage of missing data was assessed using multiple imputation and then replaced with the mean response of participants.

### Reliability

In this study, internal consistency and test–retest reliability were used:

#### Internal consistency

To evaluate the internal consistency of the family caregiver burnout construct, Cronbach’s alpha coefficients, McDonald’s omega, and composite reliability were used (56). Construct reliability or internal consistency of factors serves as an alternative to Cronbach’s alpha coefficient in structural equation modeling analysis (55).

#### Test–retest reliability

To determine the stability of the instrument, 28 family caregivers were asked to complete the final instrument twice, with a two-week interval between each completion. The intraclass correlation coefficient was calculated for all dimensions and for the entire instrument. An intraclass correlation coefficient of at least 0.4 was considered acceptable (57).

### Convergent validity

After fitting the structural model, convergent validity of the family caregiver burnout construct was evaluated using Fornell and Larcker’s (58) criteria, composite reliability (CR), and average variance extracted (AVE). Convergent validity is achieved when the items of a construct correlate highly with each other and represent their respective construct. For convergent validity to be established, AVE > 0.5, CR > 0.7, and CR > AVE must be met (58).

### Invariance testing

In this study, to test the invariance (equivalence) of the factor structure of family caregiver burnout by gender, a series of confirmatory factor analysis methods were used. First, the six-factor model was fitted separately for each gender group (male and female), and then a baseline measurement model without equality constraints was created for both groups. Invariance was tested using the chi-square difference test (Δ*χ*^2^) and the comparative fit index (ΔCFI) (59). Measurement invariance was then assessed to determine the gender invariance of the six-factor structure of family caregiver burnout (60).

### Standard error of measurement

The standard error of measurement is an indicator of the precision and reliability of the instrument. Due to the presence of error in repeated measurements, there is always some degree of variation (61). In this study, the standard error of measurement was also evaluated as a crucial psychometric property of the instrument. A lower standard error of measurement is important because clinically significant changes should be above the standard error of measurement (62). Additionally, the agreement parameter of the instrument was assessed by considering the smallest detectable change (SDC) and the minimal clinically important difference (MCID). The agreement parameter is positive if the SDC is greater than the MCID, indicating that the change is real and not due to measurement error (62).

### Ceiling and floor effects

Ceiling and floor effects occur when more than 15% of respondents achieve the highest or lowest possible score, respectively. The presence of ceiling and floor effects indicates that the instrument may not include items representing the maximum and minimum intensity of the phenomenon, suggesting inadequate content validity (63).

### Validity of the mixed-methods study

To ensure the validity of the mixed-methods study, the researcher addressed threats during data collection by using different participants in the qualitative and quantitative stages, ensuring that none of the qualitative study participants participated in the secondary quantitative study. Additionally, an adequate sample size was used in the qualitative phase, and a larger sample size was employed in the quantitative phase. To mitigate threats during data analysis, the instrument was designed based on themes and major categories extracted, and all processes during both the design and validation phases were meticulously conducted by the researcher and reviewed by advisors. To address potential threats during data interpretation, the researcher avoided comparing qualitative results with quantitative results directly but rather linked them. Following typical exploratory design procedures, the quantitative study was built on the qualitative study, and during data interpretation, qualitative data were interpreted first, followed by the interpretation of quantitative data.

### Statistical analysis

To examine EFA and perform statistical tests related to the research hypotheses, SPSS version 26 was used. For validating the construct of family caregiver burnout, CFA using first and second-order maximum likelihood estimation was conducted using AMOS version 24. Multiple fit indices were employed to assess the model fit, including the chi-square to degrees of freedom ratio (χ^2^/df), the Parsimonious Normed Fit Index (PNFI), the Comparative Fit Index (CFI), the Parsimonious Comparative Fit Index (PCFI), the Incremental Fit Index (IFI), the Goodness of Fit Index (GFI), and the Root Mean Square Error of Approximation (RMSEA) (59). Convergent validity was evaluated using Fornell and Larcker’s criteria (1981) (58). To confirm the number of factors extracted in the EFA, parallel analysis was used. Additionally, to validate the six-factor structure of this scale, a novel network analysis approach using the R software was employed.

### Ethics approval and consent to participate

Ethical considerations in this study included obtaining approval from the Ethics Committee of Babol University of Medical Sciences (Code: IR.MUBABOL.HRI.REC.1400.115), obtaining permission for audio recording, explaining the objectives and methodology to participants, securing written informed consent, informing participants of their right to withdraw at any stage of the research, adhering to the principle of non-maleficence, maintaining confidentiality and anonymity of participant information, and offering to share the research results with participants if they wished.

## Results

### Descriptive statistics

In this study, 594 caregivers of the older adults, with a mean age of 46.88 ± 10.84 years (age range: 21–80 years), were examined. Additionally, the mean duration of caregiving was 6.86 ± 6.33 years (range: 1–40 years). Other demographic variables of the participants are presented in Table 2.

**Table 2:** Demographic characteristics of family caregivers.

Variable	Levels	Total (*n* = 594)	
		Count	Percentage (%)
Gender	Female	470	79.1
	Male	124	20.9
Marital status	Single	130	21.9
	Married	418	70.4
	Widow	12	2
	Divorced	34	5.7
Relationship with older adults	Spouse	50	8.4
	Child	432	72.7
	Grandchild	48	8.1
	Daughter/Son-in-law	30	5.1
	Other Relatives	34	5.7
Education level	Below diploma	70	11.8
	Diploma	102	17.2
	University education	422	71
Income level	Sufficient	276	46.5
	Partially sufficient	196	33
	Insufficient	122	20.5
Employment status	Employee	248	41.8
	Self-employed	94	158
	Retired	76	12.8
	Housewife	146	24.6
	Unemployed	30	5.1
Number of children	None	184	31
	1	150	25.3
	2	170	28.6
	3	64	10.8
	4 or more	26	4.4
Insurance coverage	Yes	510	85.9
	No	84	14.1
Daily caregiving time	Less than 2 h	174	29.3
	More than 2 h	420	70.7
Living situation	Near the older adult	332	55.9
	Living with the older adult	262	44.1
Age (years); Mean (SD)		46.88 (10.84)	
Duration of caregiving (years); Mean (SD)		6.86 (6.33)	

### Results of designing items for the FCBI of older adults with chronic disease

#### Qualitative results

The qualitative analysis resulted in 199 codes, 17 initial categories, and six final categories. Based on findings from interviews with family caregivers, literature reviews, and tools related to occupational burnout, a pool of 228 items (29 items derived from the literature review and 199 from the qualitative analysis) was developed using a 5-point Likert scale (always, often, sometimes, rarely, and never). After several rounds of review by the research team, 126 items were deleted or merged, and the remaining 102 items proceeded to the psychometric evaluation stage.

#### Quantitative results

#### Face and content validity

At this stage, all participant feedback on the appearance of the items was considered. In the quantitative face validity assessment, two items, “Life has dealt me a bad hand” and “My marital life has collapsed” were deleted due to scores below 1.5, resulting in a 100-item tool for the next phase. During the qualitative content validity review, some items were merged, and proposed changes to the appearance of items were made. Ultimately, the tool was reduced to 60 items. During the content validity ratio assessment, four items, “I’m stuck in the ‘if only’ and ‘maybe’“, “I blame myself,” “I’m upset with myself and look for my weaknesses,” and “I’m ashamed and embarrassed by the care recipient’s behavior” were removed due to scores below 0.62 and numerical judgments below 1.5, reducing the tool to 56 items. In the content validity index assessment, two items, “I’m on alert” and “I’m embarrassed by others’ disgusted looks” were removed due to scores below 0.7. Finally, the FCBI of older adults with chronic disease, consisting of 54 items, proceeded to the construct validity assessment stage.

### Ease of use of the FCBI of older adults with chronic disease

The average time to complete the FCBI of older adults with chronic disease was 8 min (range 6–12 min). The non-response rate for all items was 0 %.

#### Construct validity

**Figure 1:**
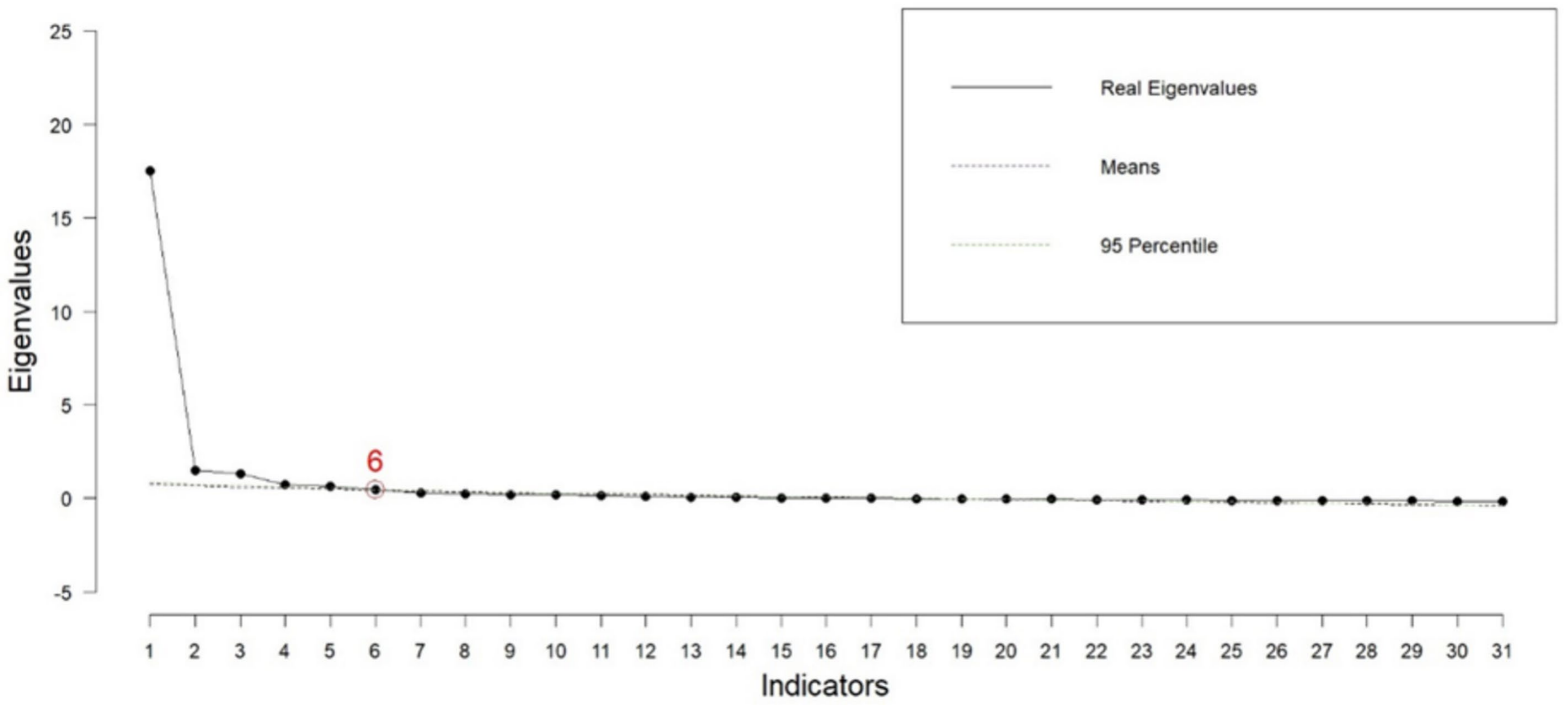
Parallel analysis for determining the number of extractable factors.

The results of the exploratory factor analysis indicated that the sample size for factor analysis was adequate, with a KMO value of 0.965, suggesting the data were sufficient for analysis. The Bartlett’s test result (*p* < 0.001, *x*^2^ = 9440.56) was significant, indicating sufficient correlations between items to justify factor analysis. The exploratory factor analysis of the FCBI revealed six factors: neurosis, feeling victimized, extreme fatigue and helplessness, neglect or abuse of self and others, adaptation deficiency, and emotional separation. These six latent factors explained 17.04, 12.54, 7.53, 5.74, 5.10, and 4.98% of the variance, respectively, accounting for a total of 52.93% of the variance in the FCBI. Twenty-three items were excluded from the analysis due to factor loadings below 0.4 and cross-loadings (see Table 3 and Figure 1).

**Table 3:** Exploratory factors extracted after promax rotation.

Factor	Item	Factor loading*	Communality**	Variance (%)	Eigenvalue
Feeling victimized	Caring has made me regret lost dreams.	0.897	0.665	12.54	4.13
	I feel victimized because my needs are lost among the many needs of the care recipient or others.	0.781	0.769		
	The pressure of caregiving has made me think constantly that I do not deserve this situation.	0.904	0.782		
	I feel that I have lost my entire future because I was a caregiver.	0.911	0.826		
	I am upset that caregiving has become my duty.	0.738	0.677		
	My caregiver identity has overshadowed my identity as a man/woman, and it upsets me.	0.729	0.761		
Extreme fatigue and helplessness	My energy for caring for myself and others is exhausted.	0.528	0.703	7.53	2.48
	I feel helpless in performing multiple caregiving tasks for the patient and others.	0.444	0.726		
	The stress from caregiving has made me lose my ability to cope with stressors.	0.472	0.714		
	The pressures of caregiving have made me dependent on others for daily tasks.	0.798	0.580		
	Caring has made me lose my confidence in meeting my personal needs.	0.543	0.739		
	Following care recipient’s or my own treatment regimens has become overwhelming.	0.676	0.749		
	I lack the ability to think and analyze caregiving or other personal matters.	0.631	0.765		
Neglect or abuse of self and others	The pressures of caregiving have led me to self-harm when I am nervous or helpless.	0.776	0.685	5.74	1.89
	I mistreat the care recipient or others (e.g., ignoring, shouting, swearing, hitting, throwing objects).	0.961	0.929		
	Caring has made me turn a blind eye to my personal needs.	0.608	0.458		
Neurosis	Caring has made me irritable.	0.586	0.619	17.04	5.62
	Caring has made me restless.	0.675	0.653		
	I cry when recalling the hardships of caregiving.	0.774	0.627		
	Caring has made me live in fear and anxiety.	0.915	0.694		
	Caring has made me feel that everything in my life is repetitive.	0.665	0.757		
	I no longer enjoy what I used to before caregiving.	0.666	0.671		
	Caring has made me lose the meaning of life and being alive.	0.739	0.677		
	Caring has made me suffer from constant worry.	0.812	0.660		
	Caring has made me feel prematurely aged.	0.793	0.690		
	Caring has made me develop various physical illnesses.	0.502	0.574		
	I am terrified at the thought of myself or other family members needing care.	0.644	0.573		
Emotional separation	Caring has made me avoid connecting with others.	0.734	0.805	4.98	1.64
	Caring has put me in a situation where I feel imprisoned.	0.750	0.851		
	After caregiving, I feel that my family or others have abandoned me.	0.763	0.828		
Adaptation deficiency	I suffer from others’ hurtful comments/jokes/judgments about myself, the care recipient, or other family members.	0.618	0.665	5.10	1.68
	I can no longer convince myself that caregiving for the patient and others is the best course of action.	0.732	0.701		
	I feel that providing care for myself or others is beyond my capacity.	0.852	0.925		

*Factor loadings obtained through promax rotation (greater than 0.4).

**Figure 2:**
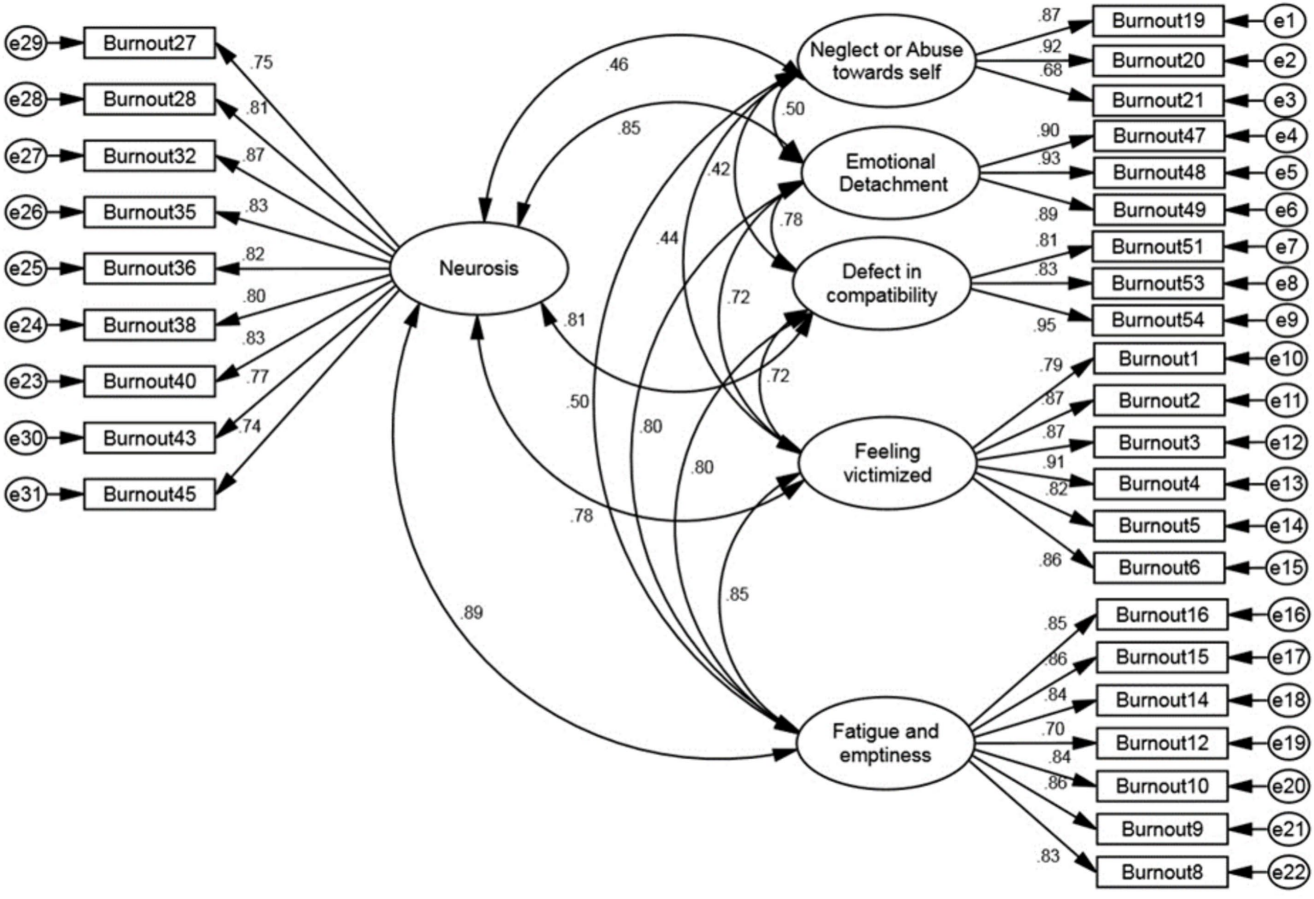
First-order confirmatory factor analysis of the family caregiver burnout construct.

The results of the CFA indicated that all fit indices—PCFI = 0.847, PNFI = 0.809, CMIN/DF = 2.23, RMSEA = 0.065, IFI = 0.941, CFI = 0.940, and GFI = 0.923—confirmed the model’s good fit. Therefore, the six-factor model of the FCBI was validated (Table 4). The standardized factor loadings between items and factors in the first-order factor analysis showed that all factor loadings in the model were above 0.4 (Figure 2).

**Table 4:** Fit indices for the CFA model of the family caregiver burnout construct.

Fit Indices CFA Model	χ^2^	Df	*p*-value	CMIN/df	RMSEA (CL90%)	PNFI	CFI	PCFI	IFI	GFI
First Order	935.31	419	<0.001	2.23	0.065 (0.059–0.070)	0.809	0.940	0.847	0.941	0.923
Second Order	968.23	428	<0.001	2.26	0.065 (0.060–0.071)	0.823	0.938	0.863	0.938	0.920

*Acceptable thresholds for fit indices: PNFI, PCFI (>0.5), GFI, CFI, IFI (>0.9), RMSEA (<0.08), CMIN/DF (3 > good, 5 > acceptable).

**Figure 3:**
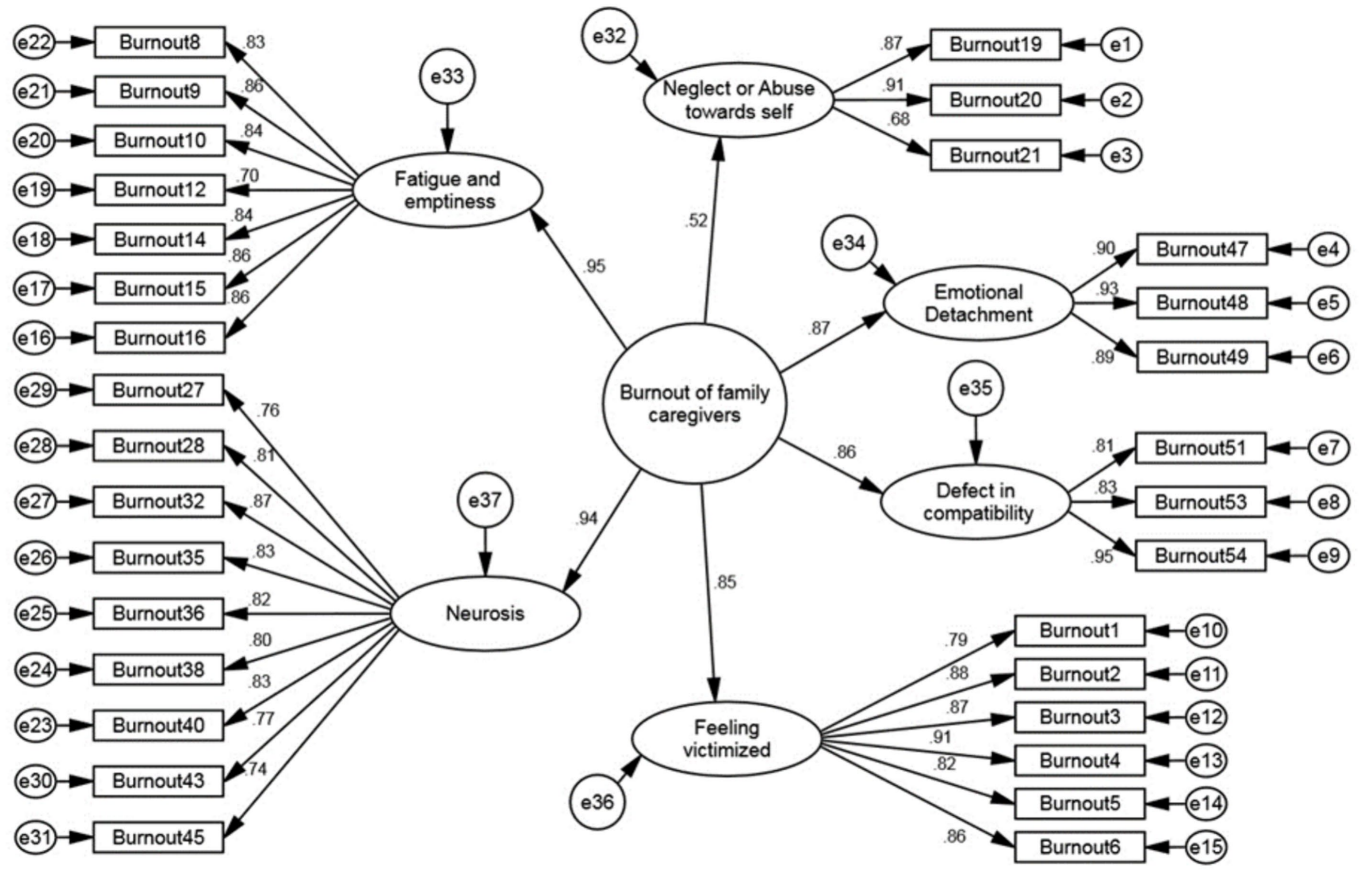
Second-order confirmatory factor analysis of the family caregiver burnout construct.

In the second-order confirmatory factor analysis, the fit indices indicated an acceptable fit of the proposed model with the data (Table 4). The correlation values between the factors of the FCBI indicated weak to moderate correlations between the factors, suggesting that the factors are divergent from one another (Figure 3).

The results of the first-order confirmatory factor analysis showed that the AVE values for the six factors of the FCBI were all greater than 0.5 and at an acceptable level. Additionally, the CR values for each factor were greater than their respective AVE values. These results indicate that the FCBI has good convergent validity (Table 5).

**Table 5:** Convergent validity, internal consistency, and stability of the family caregiver burnout construct.

Factors	*α*	Ω	CR	AVE
Neurosis	0.942	0.942	0.942	0.645
Feeling victimized	0.942	0.942	0.942	0.732
Extreme fatigue and helplessness	0.938	0.940	0.938	0.686
Neglect or abuse of self and others	0.841	0.842	0.865	0.685
Adaptation deficiency	0.892	0.896	0.897	0.746
Emotional separation	0.931	0.931	0.931	0.818
Total	0.975	0.976	0.935	0.713

*α, Cronbach’s alpha coefficients; Ω, McDonald omega coefficient; CR, construct reliability; AVE, average variance extracted.

#### Reliability

The results of internal consistency for the family caregiver burnout construct showed that the internal stability of the factors was greater than 0.7 (Table 5).

#### Invariance testing

The fit indices for the model separated by gender indicated that the six-factor model had an acceptable fit in each gender group (Table 6).

**Table 6:** Results of multigroup confirmatory factor analysis in different subgroups.

Model	*χ*^2^	df	*χ*^2^/df	CFI	GFI	RMSEA	ΔCFI	ΔRMSEA
**Multigroup comparison**								
Unconstrained model	1862.79	838	2.22	0.948	0.921	0.045		
Model with factor loadings constrained	1891.09	863	2.19	0.947	0.920	0.044	0.001	0.001
Model with structural covariances constrained	1893.67	884	2.14	0.944	0.918	0.043	0.004	0.002
Model with measurement residuals constrained	1907.23	915	2.08	0.940	0.926	0.042	0.008	0.003

Fit indices: CFI, GFI (>0.9), RMSEA (<0.08), CMIN/DF (<3 good, <5 acceptable).

#### Network analysis

**Figure 4:**
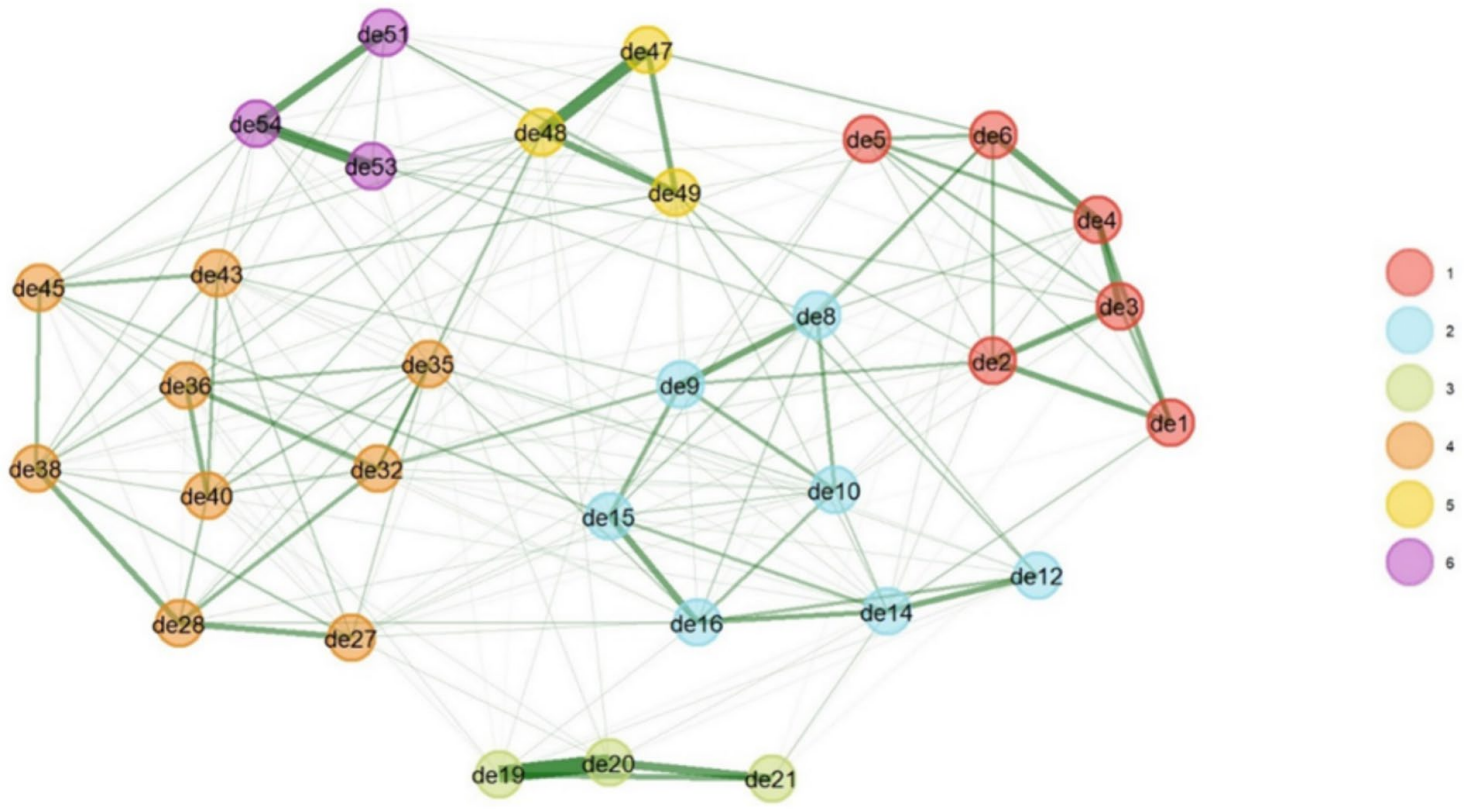
Network analysis results for the family caregiver burnout construct.

Network analysis estimated the six-factor structure of the family caregiver burnout construct. All item dimensions were similar to the findings of the exploratory factor analysis, as shown in Figure 1. Based on the bootstrap test and 95% confidence interval with a standard error of 0.063, the median of the six dimensions was obtained (Figure 4).

### Scoring the FCBI of older adults with chronic disease

The FCBI uses a Likert scale ranging from one to five (Always = 5, Often = 4, Sometimes = 3, Rarely = 2, Never = 1). The score range is from 31 to 155. A score of 1 to 31 indicates no burnout, 32 to 73 indicates mild burnout, 74 to 114 indicates moderate burnout, and 115 to 155 indicates severe burnout.

## Discussion

This exploratory mixed-methods research aimed to design and psychometrically evaluate the FCBI of patients with chronic disease and consisted of two sequential qualitative and quantitative phases. In sequential mixed-methods approaches, qualitative and quantitative data are interconnected (64). In the present study, a Likert scale ranging from one to five was used to score the FCBI of patients with chronic disease. The frequency-based Likert scale was deemed the most suitable for assessing family caregiver burnout, as the intensity may vary across different items (65). The nature of Likert scales is generally considered ordinal (66). Items based on the Likert scale can be three, four, five, six, or seven options, with the five-point scale being the most optimal (66).

In the quantitative face validity assessment, the impact score of each item was calculated. This method involves using respondents’ feedback to reduce and eliminate unsuitable items and to determine the importance of each item (67). Qualitative content validity was evaluated with the assistance of 10 experts familiar with the instrument’s constructs through the calculation of the CVR and CVI. Many researchers recommend using 10 or more experts for content validity assessment (68, 69). Subsequently, quantitative content validity was assessed using both the CVR and CVI methods with the same experts. Although many studies only mention qualitative content validity assessment (45), it is important to note that this approach is not methodologically flawless. Therefore, it is recommended that content validity be calculated quantitatively based on experts’ opinions using both CVR and CVI indices (70, 71).

In this study, construct validity was assessed using exploratory and confirmatory factor analysis. Factor analysis, which identifies the dimensions of an instrument, is part of construct validity, referred to as structural validity by Mokkink and colleagues (72). Before conducting the EFA, the correlations between items were examined to determine whether the instrument was unidimensional or multidimensional. This dimensionality was then confirmed through CFA (63). Once the subscales were identified, internal consistency for each subscale was calculated. An internal consistency between 0.7 and 0.9 is considered appropriate (73). Before extracting factors, the KMO test and Bartlett’s test of sphericity were performed to ensure sample adequacy and verify that the items were suitable for principal component analysis (74). The results indicated that the sample size for factor analysis was excellent, and Bartlett’s test was significant, confirming sufficient correlations between items for factor analysis. KMO values between 0.7 and 0.8 are considered good, and values between 0.8 and 1 are considered excellent (75).

In this study, EFA and CFA were each conducted with a sample of 297 participants (total 594 participants). Sample size is crucial in factor analysis, with a minimum of 100 participants generally recommended (59). Hatcher and O’Rourke (76) recommend at least five samples per item. According to some researchers, a sample size of 200 to 300 is sufficient for factor analysis (77, 78). Costello and colleagues suggest the best approach to determine sample size is the sample-to-item ratio, recommending 10 to 20 samples per item (79). EFA was performed using principal component analysis and varimax rotation. Collecting data online minimized missing data, which were then addressed using multiple imputation and replaced with the mean response of participants. Another important consideration during factor analysis is the acceptable level of communalities and the variance explained by each factor and the total variance explained (80). The type of rotation used should also be specified, as rotation simplifies and clarifies the structure (79). In this study, after confirming sample adequacy, the number of factors was determined using scree plot and eigenvalue methods. The results showed that the family caregiver burnout construct comprised six factors: “neurosis,” “feeling victimized,” “extreme fatigue and helplessness,” “neglect or abuse of self and others,” “adaptation deficiency” and “emotional separation” which together explained 52.93% of the total variance in the family caregiver burnout construct. At this stage, 23 items were excluded due to factor loadings below 0.4 and cross-loadings, resulting in a final 31-item instrument.

### First factor: feeling victimized

This factor comprises six items that measure the caregiver’s feelings about the outcomes of caregiving, such as regret, feeling victimized, having no future, and losing identity. This factor accounts for the largest percentage of variance. In the MBI, the item “At the end of a workday, I feel like I’ve been used up” also measures feeling victimized (81). To explain this finding, it can be stated that many family caregivers claim they face two primary responsibilities: their personal lives and caregiving for the older adults. Consequently, caregiving can disrupt their personal and professional plans. Often, they prioritize the needs of the care recipient over their own, leading to disruptions in their daily routines and loss of personal goals. This issue is more prevalent when caring for older adults with Alzheimer’s, dementia, or stroke, which require more demanding and time-consuming care (82). Peacock et al. (10) described this situation as “life on hold” for caregivers, as they have to take leave, request job transfers, or even leave their jobs. Faronbi et al. (83) stated that caregiving prevents caregivers from attending daily or family events and fulfilling other personal responsibilities. Additionally, they are in a state of compulsion, often lacking enough time to spend with their families or to pursue career advancements. This state of compulsion fosters feelings of victimization. Feeling victimized reduces acceptance and adjustment to the caregiver role. Caregivers, in fact, become hidden patients who, due to their caregiving responsibilities, may be unable or unwilling to take care of their own health needs (84). Goodman and Punoos refer to long-term family caregivers as the “second victim” and the families of individuals with chronic disease as the “potential patient” (85).

### Second factor: extreme fatigue and helplessness

This factor includes seven items and measures feelings of instability, feelings of helplessness, excessive fatigue, and dependency on others for making caregiving decisions and performing daily life activities. One of the items in the Tamarana et al. (28) ICBS is “I feel tired when I spend time caring for the recipient”. In the 10-item ICBI by James Nicholas, this factor ranked third in terms of factor loading (0.809), with six items measuring fatigue in informal caregivers: “I always feel tired,” “I feel emotionally drained,” “I feel physically drained,” “Caregiving is physically exhausting,” “Caregiving is emotionally exhausting” and “I feel burned out from caregiving” (27). The 17-item OLBI also includes an eight-item dimension that measures fatigue (86). The MBI identifies this factor as one of the critical dimensions for measuring burnout (81).

### Third factor: neglect or abuse of self and others

This factor includes three items and measures neglect and abuse toward oneself and the care recipient. One of the items in the Tamarana et al. (28) ICBS is “I get angry at the care recipient’s demands” which is used to measure symptoms of burnout. Abuse by caregivers can be physical, financial, sexual, emotional, or psychological. In older adults, psychological abuse is more prevalent and includes emotional mistreatment, verbal abuse, deprivation of contact, humiliation, blaming, and controlling behavior by the caregiver. It also includes intentional or unintentional neglect, financial exploitation, and abandonment (87). The study by Ashrafzadeh et al. (88) found that burnout is associated with symptoms such as failure to follow the treatment regimen carefully, leaving the patient alone at home, verbal aggression in response to repeated questions, neglecting personal hygiene protocols, abandoning the patient, and not paying adequate attention to the needs of the patient under their care.

### Fourth factor: neurosis

This factor includes 11 items and measures the psychological and physical consequences of caregiving. One of the items in the James Nicholas ICBI is “I often feel hopeless” (27). Two items in the Tamarana et al. (28) ICBS include “My physical health has deteriorated because of caregiving responsibilities” and “My sleep is affected by my caregiving responsibilities” which also measure symptoms of neurosis in caregiver burnout. To explain this finding, it can be said that the majority of older adults with chronic diseases, especially those with Alzheimer’s, are cared for at home. The burden of caring for such patients particularly falls on relatives who try to support and care for them (89). This is why these caregivers are often referred to as “hidden patients” (90), as providing care for an older adult, vulnerable person can expose the caregiver to negative physical, emotional, and social consequences (89). Caregivers frequently struggle with a world of worries, fears, and anxieties, along with their internal turmoil, which may inadvertently affect the health of the care recipient as well (91–93).

### Fifth factor: emotional separation

This factor includes three items and measures aspects such as ineffective communication and feelings of social isolation. According to numerous studies, burnout is associated with symptoms such as feelings of loneliness, isolation, boredom, and frustration among caregivers (94–98). Caregivers often suffer from emotional loneliness (e.g., lack of intimacy) and social loneliness (e.g., absence of an extensive social network) (99). Generally, social isolation in these individuals occurs in two main dimensions: structural (e.g., infrequent or rare social contacts, limited social interactions, and poor participation in social activities) and functional (e.g., lack of a sense of belonging or dissatisfaction with social relationships), where functional isolation is closely linked with social loneliness (100–102). Social isolation and feelings of loneliness and abandonment have been identified as risk factors for both physical and mental health (103).

### Sixth factor: adaptation deficiency

This factor includes three items and measures the consequences of the inability to adapt to caregiving situations, such as addiction, dissatisfaction with life and current conditions, and the inability to care for oneself and others. One of the items in the Tamarana et al. (28) ICBS is “I compromise my responsibilities regarding self-care”. A critical aspect of caregiving often overlooked by caregivers is their coping strategies. These coping strategies, ranging from resilience and optimism to despair and withdrawal from providing care, play a vital role in determining how caregivers adapt to their multiple roles as caregivers. Coping strategies can significantly modulate the level of caregiving burden, either amplifying or mitigating it (104, 105). Notably, the nature and extent of the burden caregivers feel and its consequences, such as burnout, depend on their unique coping strategies, belief systems, and attitudes (91).

After conducting EFA, CFA was performed in this study. The results of the model fit evaluation for the six-factor model of family caregiver burnout showed that all indices confirmed a good fit for the model (59). Among burnout instruments, the MBI and its various versions have been used extensively by researchers for structure, development, and measurement of burnout (26, 106). The MBI is considered the gold standard for measuring occupational burnout (107) and is used in over 90% of studies in the field of burnout syndrome (106). However, this has led to a close link between theory and measurement, ultimately “neglecting other conceptual approaches to burnout” (106). Additionally, the scoring of the three dimensions of this scale in both positive and negative ways has led to artificial clustering of sub factors (108). On the other hand, results from extensive factor analysis on 45 studies using the MBI showed that in addition to the three-factor model by Maslach, empirical data also support alternative models, including two-, four-, or five-factor models or models with higher factors (109).

Although the CBI (26) and the Shirom-Melamed Burnout Measure (SMBM) were developed to address this issue, reducing the burnout tool to a unidimensional structure has been strongly discouraged by several researchers (107, 110, 111). The OLBI was proposed as an alternative to address the content and methodological drawbacks of the aforementioned occupational burnout tools (112, 113). However, the items in these tools are specifically related to work and education and cannot cover burnout caused by caregiving by informal caregivers. Therefore, the ICBI (27) and the ICBS (28) were designed for family caregivers, but both have a unidimensional structure. Hence, there is still a need for a valid and multidimensional tool to measure burnout, especially in different target groups (114).

Given the aforementioned points, the tool designed in the present study (using both deductive and inductive methods) specifically measures the symptoms of burnout among family caregivers of older adults with chronic diseases and comprises six factors, covering the deficiencies present in available tools. The tool designed in this study also has acceptable reliability. The most common method for examining internal consistency is the calculation of Cronbach’s alpha. For multi-option Likert scales, Cronbach’s alpha is used to determine internal consistency (61, 72). In this study, Cronbach’s alpha coefficients, McDonald’s omega, and composite reliability were used to evaluate the internal stability of the family caregiver burnout factors. The results showed that the internal consistency of the burnout construct for family caregivers of older adults with chronic diseases was acceptable. Reliability greater than 0.7 was considered appropriate (60).

The results also showed that the convergent validity of the six factors of the family caregiver burnout construct was at an acceptable level, with high correlations among items within a factor, indicating that they represented their respective constructs well. Thus, this tool has acceptable convergent validity, which is a part of construct validity and helps establish the tool’s credibility (61).

The final tool in this study includes 31 items, with at least three items per factor. There is no specific rule for the number of items that should be extracted. Instruments with fewer items have lower response errors (due to less fatigue among respondents), and the items should represent the intended content. To achieve the desired internal consistency, having at least three items per construct is necessary (37).

This research is the first study to design a domain-specific tool for measuring burnout among family caregivers of older adults with chronic diseases. As such, it provides a foundation for conducting similar studies in different cultures with adequate sample sizes. Although the label of this tool includes the term “older adult” due to the researcher’s focus as a geriatric nursing student, the items are not specific to the older adult group. Therefore, this tool can be used to assess burnout among family caregivers of adult patients with chronic diseases. This study offers a stronger theoretical foundation for research on family caregiver burnout. The use of both inductive and deductive approaches for item design is another strength of the present study. Most designed tools have only focused on assessing the validity of the tool. They have mainly relied on internal consistency and, occasionally, stability, while other aspects of reliability, such as standard error and reproducibility, have been overlooked. In this study, we evaluated most reliability indices. This study also had limitations. For instance, due to limited access to male caregivers in the research environment, we could not include more male caregivers in our study. Divergent validity was not assessed in this study. Completing the tool through self-reporting and the potential impact of the physical, emotional, and psychological state of the participants on their responses to the items is another limitation of the present study. Another limitation relates to the nature of the convenience sampling method used in the construct validity section. The descriptive-cross-sectional study conducted to assess construct validity was performed in a specific geographic area, which may limit the generalizability of the results. The use of online data collection methods might affect the validity of the data, as those with access to the internet may not be representative of the general population of family caregivers of older adults with chronic diseases.

## Conclusion

The results showed that the FCBI has acceptable validity and reliability. It consists of 31 items for assessing burnout among family caregivers and includes 16 demographic items, totaling 47 items. Consequently, it takes approximately 6 to 12 min to complete. In designing this tool, efforts were made to ensure both appropriate content validity and a manageable number of items so that its use would not be time-consuming or exhausting for nurses and other healthcare providers. The items of this tool have been revised at various stages based on the feedback from experts and users, resulting in items that are understandable and acceptable to healthcare providers and users. Therefore, this tool can be used as an easy-to-respond, straightforward, and appropriately itemized instrument for measuring burnout among family caregivers. It is recommended to use this tool during follow-up visits in clinical centers or home visits in the community by healthcare providers, especially nurses.

## Data Availability

The raw data supporting the conclusions of this article will be made available by the authors, without undue reservation.
